# 

**Published:** 1874-12

**Authors:** 


### TABLE OF CONTENTS.

### ORIGINAL COMMUNICATIONS.

ANEURISM OF THE FEMORAL ARTERY, TREATED BY DIGITAL COMPRESSION—CURE. *By James McCann, M.D., Pittsburgh, Pa* .......... 513

ANTEVERSION OF THE UTERUS OF FOUR YEARS STANDING—REDUCTION. *By J. H. Lowe, M.D., Atlanta, Ga* .......... 521

CLINICAL LECTURE ON LIPOMA, CANCER AND ESMARCH'S TOURNIQUET. *By Prof. W. F. Westmoreland, M.D., Atlanta, Ga* .......... 522

### ATLANTA ACADEMY OF MEDICINE.

Supposed Extra-Uterine Fœtation—Ipecac Treatment of Dysentery—Gonorrhœal Orchitis—Hydro-Metra—Triple Pregnancy with Hydro-Metra—Milk Leg; its Pathology—Views of Dr. Coe—Anomalous, Rhumatoid Disease of the Feet .......... 527

### OUR EXCHANGES.

The Kings and Slaves of Business .......... 538

Guaiacum in Acute Tonsillitis ..........

Spermatic Colic .......... 555

Is Smoking a Sin?—Mr. Spurgeon's opinion .......... 556

Solvent for Gall-Stones ..........

Pruritus Vulvæ, Treatment .......... 557

Cauterization of Cavity of the Uterus .......... 558

Castor Bean, Cultivation ..........

The "Man of the Fork" ..........

Emulsions, Preparation of .......... 559

Cases of Cremation ..........

Mirabile Dictu!! .......... 561

Mustard Blisters .......... 564

External Use of Chloral in Cancer ..........

Stammering, Treatment .......... 565

Cough—Experimental Study .......... 566

Removal of Mucus from Cervical Canal ..........

Explosive Medicines ..........

Blood Letting .......... 567

The Montpellier Oath ..........

To Keep Cider Sweet ..........

Catarrh Snuff .......... 568

### BIBLIOGRAPHICAL.

Stille's Materia Medica .......... 569

Tyson on Examination of Urine ..........

Sir H. Thompson on Urinary Organs ..........

Davis' Clinical Lectures .......... 571

### EDITORIAL.

Embryotomy—Novel Expedient .......... 572

Status of Medical Corps of the Army .......... 574

Excursion to the Mediterranean ..........

Atlanta Quackery .......... 575

Trouble in Arkansas Medical Association ..........

Amende .......... 576

